# Genetic Diversity Analysis and Polyploid Induction Identification of *Idesia polycarpa*

**DOI:** 10.3390/plants13233394

**Published:** 2024-12-03

**Authors:** Xiaomei Luo, Yunke Liu, Yuting Lei, Zhoujian He, Xiao Gong, Meng Ye, Qiangang Xiao

**Affiliations:** 1 National Forestry and Grassland Administration Key Laboratory of Forest Resources Conservation and Ecological Safety on the Upper Reaches of the Yangtze River, College of Forestry, Sichuan Agricultural University, Chengdu 611130, China; mihualyt@126.com (Y.L.); hezhouj@163.com (Z.H.); gongxiao202209@163.com (X.G.); yemeng5581@163.com (M.Y.); 2 Chengdu Academy of Agriculture and Forestry Sciences, Nongke Road 200, Wenjiang District, Chengdu 611130, China; lykchengdu@sina.com (Y.L.); xiaoqg1992@163.com (Q.X.)

**Keywords:** genetic diversity, breeding parent, polyploid induction, ploidy identification

## Abstract

*Idesia polycarpa* from Sichuan is a valuable germplasm with high economic potential, but it faces variety scarcity. To address this, this study collected 16 varieties (lines), identifying IpHT1 as a promising parent due to its high oil content (38.5%) and red fruits. Polyploid induction via adding 0.50% colchicine to Murashige and Skoog (MS) medium yielded 520 IpHT1 mutagenized seedlings. Subsequently, flow cytometry (FCM) was performed on 401 morphologically variant seedlings which had been initially screened, resulting in the identification of 15 suspected triploids, 35 suspected tetraploids, and 3 chimeras. Furthermore, fluorescence in situ hybridization (FISH) analysis found that the probe (AG_3_T_3_)_3_ had terminal signals at both ends of each chromosome, allowing for the counting of 42 chromosomes in diploids and 84 in tetraploids. The probe 5S rDNA showed 2, 3, and 4 hybridization signals in the interphase nuclei of diploid, triploid, and tetraploid cells, respectively, but the probe (GAA)_6_ failed to produce any signal on *I. polycarpa* chromosomes. Ultimately, 18 polyploids were selected, including 7 triploids and 11 tetraploids. Triploids and tetraploids showed significant leaf morphological and physiological differences from diploids. Consequently, this study successfully established a polyploid breeding system for *I. polycarpa*, thereby enhancing its genetic diversity and breeding potential.

## 1. Introduction

*Idesia polycarpa* Maxim., belonging to the Flacourtiaceae family, is a deciduous tree native to China’s central and southern regions, Japan, Korea, and the Nansei Islands. It has been introduced to New York and both northern and southern New Zealand. The genus *Idesia* Maxim. contains only one accepted species, *I. polycarpa* [1], with three varieties [2]. Furthermore, *I. polycarpa* contains 42 chromosomes [3] and has a genome size of approximately 1.21 Gb [4]. The flowers of *I. polycarpa* are unisexual and dioecious or polygamous. Specifically, the male flowers are slightly larger than the female flowers [2,5]. Typically, *I. polycarpa* reproduces sexually, but it can also reproduce asexually [6]. As an important woody oilseed economic tree and ornamental plant, *I. polycarpa* also features fruit rich in unsaturated fatty acids, which help lower blood cholesterol [7]. Additionally, the oil extracted can prevent cardiovascular diseases and be used in lubricants, biodiesel, and aviation fuels [8]. Moreover, the protein-rich oil meal left after extraction can be used as feed and fertilizer [9]. The tree’s layered crown and colorful fruits make it an attractive ornamental, while its fast growth and high-quality timber offer significant exploitation potential [10,11]. However, as industrial planting of *I. polycarpa* expands, high-quality seedling cultivation becomes crucial [12,13]. For instance, Sichuan Province in China has only two recognized elite varieties, ‘Zaofeng No. 36’ and ‘Haitong No. 1’ [14]. Consequently, the scarcity of elite varieties and widespread unselected planting underscore the urgency of breeding. During breeding, the frequent introduction of species from other regions has led to resource overlaps and confusion in variety names. Interspecific crosses have been made and different species have been included in mixed species plantings, hindering selection and breeding efforts. Therefore, clarifying resource status is crucial for the industry’s development.

Moreover, during the breeding of forest trees, introduction and cultivation may lead to synonymy and homonymy. Hence, analyzing genetic diversity and understanding germplasm relationships can improve breeding efficiency. Phenotypic traits are commonly used to assess genetic diversity [15], as seen in studies on *Populus* L. [16], *Aquilaria sinensis* (Lour.) Spreng [17], and *Citrus reticulata* Blanco [18]. Similarly, research on *I. polycarpa* is abundant. Zhang et al. [19] found genetic differences among *I. polycarpa* provenances by observing growth indicators. Wen et al. [8] studied fruit growth and oil accumulation factors in four varieties. Feng et al. [20] discovered genetic variation in fruit-related traits, positively correlated with yield. Dun et al. [21] investigated clonal seedlings of *I. polycarpa* ‘Exuan 1’ via tissue culture. Sun [22] analyzed diurnal photosynthetic variation in tung forests. Liu et al. [23] reported variations in leaf functional traits and nutrients among provenances. Yin and Su [24] compared leaf epidermal micromorphology between sexes. Therefore, *I. polycarpa* fruit traits can serve as breeding indicators, facilitating the selection of advantageous materials [25].

However, phenotypic trait studies in plants are influenced by environmental factors and have long cycles, limiting their use in distinguishing varieties and analyzing genetic relationships [26]. Therefore, to better understand the *I. polycarpa* germplasm, the use of molecular marker techniques is necessary. Currently, these techniques are widely used in genetic diversity analysis, germplasm identification, and core germplasm bank construction. Zuo et al. [4] identified sex determination markers for *I. polycarpa*. Dai et al. [27] established an Inter-Simple Sequence Repeat-Polymerase Chain Reaction (ISSR-PCR) system for *I. polycarpa*. Wang et al. [28] found genetic variation using ISSR markers among *I. polycarpa* var. *vestita* distribution areas. Dong et al. [29] developed gender-related ISSR markers. However, ISSR markers lack stability. Consequently, Li et al. [30] and Qiu et al. [31] developed Simple Sequence Repeat (SSR) markers using transcriptome and Specific-Locus Amplified Fragment Sequencing (SLAF-seq) technology, respectively. SSR markers offer better repeatability, higher polymorphism [32], and are advantageous in variety discrimination. Thus, DNA-level research will facilitate parent selection and resource protection in *I. polycarpa* breeding.

Furthermore, polyploidy plays a key role in plant evolution and is valuable for forest tree breeding [33], enhancing fruit quality [34], stress resistance [35], and wood quality [36]. Natural polyploids are rare, but artificial induction using mutagens like colchicine and trifluralin has been successful in various plants [37,38], such as *Platanus* L. [39], *Robinia pseudoacacia* L. [40], *Populus* L. [41], *Eucalyptus urophylla* S.T. Blake [42], *Morus mongolica* (Bureau) C. K. Schneid. [43], and *Solanum muricatum* Aiton [44]. Recently, research has focused on culturing materials on mutagen-containing media, minimizing damage and enhancing induction rates, as exemplified in *Selenicereus undatus* (Haw.) D.R. Hunt [45], *Platanus acerifolia* (Aiton) Willd. [36], and *Passiflora edulis* L. [46], although this is less applied in forestry. Whlie *I. polycarpa* tissue culture techniques have been established [47], they are not for polyploid breeding. Identifying variant plants after mutagenesis involves morphological [48], cytomorphological [49], flow cytometry [50] chromosome counting [51], and rDNA loci analysis [52]. Chromosome counting is accurate but challenging, while FISH telomere probes can help determine chromosome numbers [53].

Compared to traditional methods for ploidy identification, FCM and FISH offer higher throughput and accuracy. As polyploid breeding techniques advance, rapid identification of polyploid materials from mutagenized samples is crucial. Single methods have limitations but combining them enables quick and precise selection. Unfortunately, domestic and international research on *I. polycarpa* polyploid induction and identification is lacking, hindering genetic improvement. Therefore, this study collected 16 *I. polycarpa* samples from Sichuan, using SSR to analyze genetic backgrounds and screen parents. Chromosome doubling was induced with colchicine and trifluralin. Polyploid identification involves morphological screening, FCM, and FISH. Identified polyploids are analyzed for morphological and physiological traits. Ultimately, results provide insights for breeding material selection and variety identification, promoting *I. polycarpa* polyploid breeding and elite variety cultivation. This also has implications for related families and genera.

## 2. Results

### 2.1. Genetic Diversity

**Figure 1:**
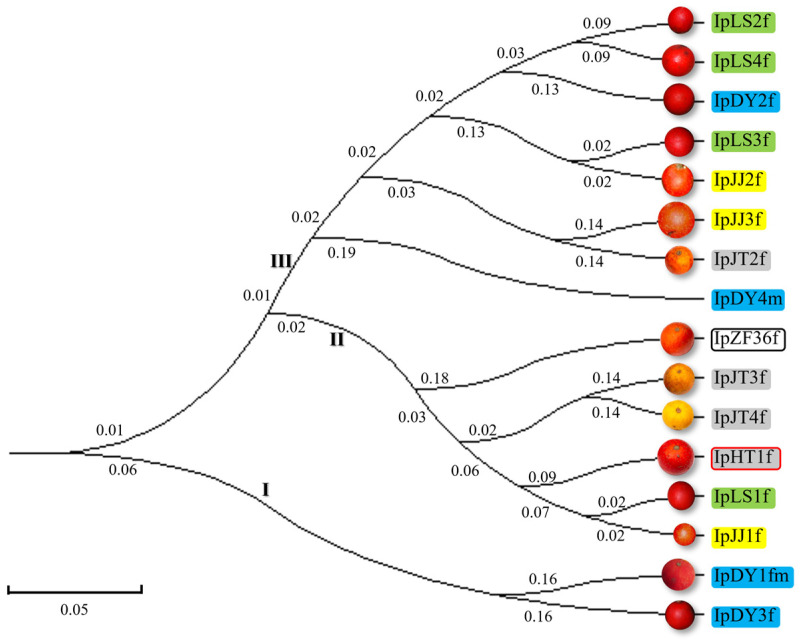
The UPGMA cluster analysis of 16 *I. polycarpa* samples revealed three distinct branches (I, II, and III) based on SSR genetic distances. Each accession’s fruit is depicted to the left, colored by origin within Sichuan Province: green for Leshan, blue for Dayi, yellow for Jiajiang, gray for Jintang, and white for Chongzhou. Accessions were named consistently: ‘Ip’ for *I. polycarpa*, with ‘f’, ‘m’, and ‘fm’ indicating female, male, or monoecious plants. The red and black frames represent two varieties, while the other lines The numbers on each branch represent the branch lengths. The distance scale is 0.05.

Polyploids inherited genetic traits from diploid parents. To obtain superior polyploids, it is important to select parental materials with good economic traits. Table 1 and Figure 1 present the characteristics of 15 *I. polycarpa* fruits, including monoecious IpDY1, male IpDY4, and 14 female plants. Moreover, fruit colors varied widely among these varieties. Specifically, Jintang County fruits showed significant diversity, including red IpHT1, orange-red IpJT2, orange-yellow IpJT3, and yellow IpJT4. Most others were red, except dark red IpDY1. Additionally, oil content ranged from 31.3% to 38.9%, with IpJJ1 (38.90%), IpHT1 (38.50%), and IpJT2 (38.06%) featuring the highest percentages. Furthermore, fruit size also differed, with IpJJ3, IpHT1, and IpZF36 featuring the largest fruit. Importantly, oil content directly impacted yield, a key evaluation criterion. As an ornamental tree, fruit color was crucial; IpHT1’s red fruits enhanced the landscape in summer and autumn. Therefore, IpHT1 combined high oil content with ornamental value.

To further explore IpHT1′s genetic background, 19 SSR primers were used on 16 *I. polycarpa* samples. After screening, 11 polymorphic primers (marked with asterisks in Table S1) were identified. The genetic parameters are presented in Table 2. Across these 11 loci, 38 alleles (Na) were detected, averaging 3.455 per primer pair (range: 2–8). Notably, Ip07 amplified the most alleles (8). The effective number of alleles (Ne) averaged 2.784 (range: 2.000–5.120), suggesting a uniform allele distribution. Furthermore, Shannon’s Information Index (I) averaged 1.033 (range: 0.693–1.836), indicating significant population variation and genetic diversity. Observed heterozygosity (Ho) was 1 for all loci, while expected heterozygosity (He) averaged 0.604 (range: 0.500–0.805), suggesting heterozygote excess and low inbreeding. Polymorphic Information Content (PIC) averaged 0.613 (range: 0.419–0.827), with 9 highly polymorphic (PIC > 0.5) and 2 moderately polymorphic primer pairs. These genetic diversity parameters reflect abundant genetic variation among the 16 samples.

Additionally, the genetic distances between the 16 *I. polycarpa* samples ranged from 0.045 to 0.591 (Table 3), indicating significant genetic variation. Close genetic relationships were observed between IpLS1-IpJJ1 and IpDY3-IpJJ2, despite IpDY3 and IpJJ2 originating from different cities. Conversely, IpLS2-IpJT2 and IpZF36-IpJT2 showed substantial genetic differences. IpJT1 had genetic distances of 0.275 to 0.545 from other samples. A UPGMA dendrogram (Figure 1) grouped the samples into three clusters: Cluster I (IpDY1 and IpDY3 from Chengdu), Cluster II (IpLS1 and IpJJ1 from Leshan, and five from Chengdu), and Cluster III (eight materials, mostly from Leshan but including IpDY2, IpDY4, and IpJT2 from Chengdu). Materials from the same origin tended to cluster, but exceptions occurred, like IpJT2 (Chengdu) and IpJJ3 (Leshan). Finally, fruit colors varied significantly, with yellow and orange in Cluster II and orange-red in Cluster III. The monoecious plant (IpDY1fm) and male plant (IpDY4m) were in different clusters, showing no clear correlation between geographical origin, fruit color, and gender.

**Figure 2:**
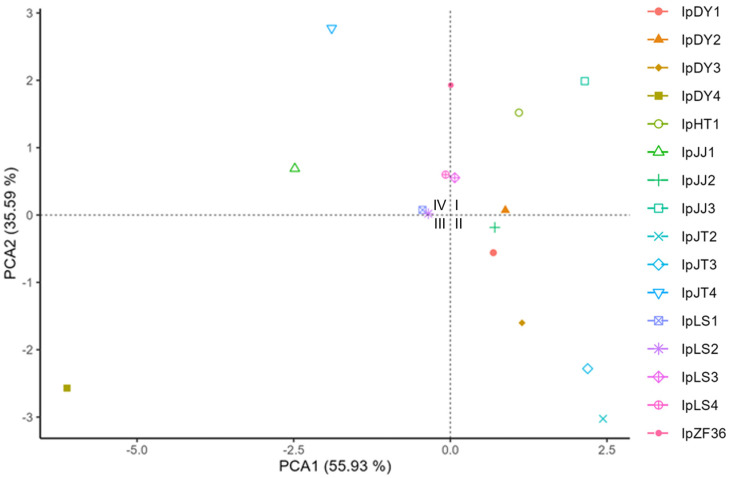
PCA cluster analysis of 16 samples of *I. polycarpa* based on fruit oil content, fruit three diameters, and SSR genetic distance.

Based on fruit oil content, fruit three-diameter (Table 1), and SSR genetic distances (Table 3), we further conducted a PCA clustering analysis, as shown in Figure 2. PCA1 and PCA2 together account for 91.52% of the total contribution, potentially providing a good reflection of the impact of different factors on the *I. polycarpa* samples. The elite varieties IpHT1 and IpZF36 are both located in Quadrant I at the top right, which may indicate that species traits located in this quadrant are relatively superior. IpJJ3, which has the largest fruit three-diameter, is also positioned in Quadrant I. The top three varieties in terms of oil content, IpJJ1, IpHT1, and IpJT2, are situated in Quadrant IV, Quadrant I, and Quadrant II, respectively, with relatively large distances between each other. Additionally, the male plant IpDY4 is located in the bottom left corner of Quadrant III, relatively distant from other *I. polycarpa plants*. IpDY1, a monoecious plant, is positioned near the center of Quadrant II, close to other female plants.

In conclusion, the selection of breeding materials was the first step in polyploid breeding. Based on the research results of genetic diversity among 16 *I. polycarpa* varieties (or lines) from different regions of Sichuan Province, it can be seen that IpHT1 exhibits significant genetic background differences from the other 15 varieties (or lines), demonstrating good specificity.

### 2.2. Identification of the Varieties and Lines

To investigate IpHT1′s molecular specificity, we first examined the allele and peak sizes of 11 SSR loci, as shown in Figure S1. Specifically, allele sizes ranged from 113 to 289, with peak sizes from 710 to 51,670. Notably, most alleles (80%) were 140–190, and most peaks (80%) were 5000–30,000. Furthermore, four SSR primers (Ip06, Ip07, Ip11, and Ip18) effectively identified 16 *I. polycarpa* samples, as illustrated in Figure S2. Subsequently, Figure S3 demonstrated identification using these primers based on allele sizes; remarkably, 13 materials were identified by a single primer. For instance, Ip18 identified IpJJ3 at an allele size of 144, while Ip11 specifically identified IpLS4 at an allele size of 200. Similarly, Ip06 distinguished IpDY1, IpDY3, and IpHT1 at allele sizes of 187, 186, and 184, respectively. Ip07 was particularly versatile, identifying IpDY1, IpDY2, IpDY3, IpDY4, IpJJ1, IpJJ2, IpJT2, IpJT3, IpJT4, and IpZF36 at various allele sizes of 211, 195, 240, 135, 139, 201, 289, 271, 113, and 149. After the initial 13 materials had been distinguished, the remaining 3 materials required Ip11 at specific allele sizes for identification. Specifically, these were IpLS1 at 185, IpLS2 at 175, and IpLS3 at 188. To summarize primer use for the 16 samples, Table 4 was compiled, omitting allele sizes for brevity but providing a clear overview.

**Figure 3:**
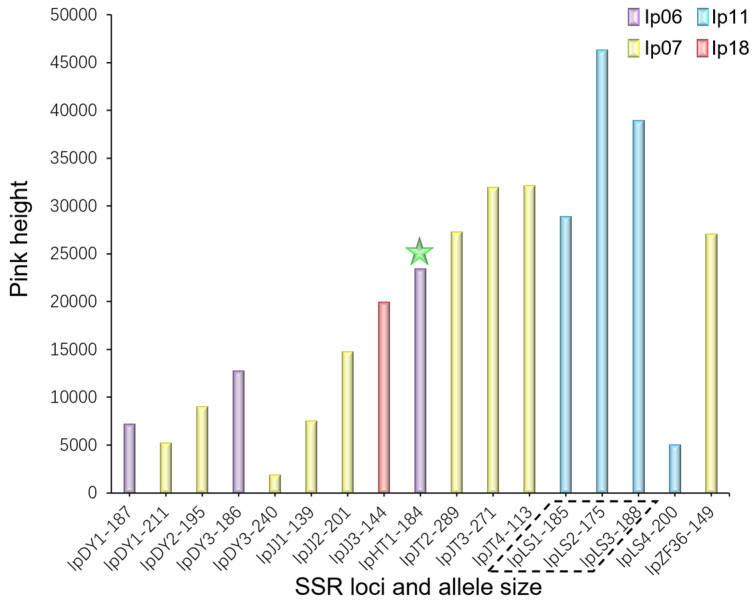
Sixteen accessions were identified by four SSR allele sizes. The horizontal axis showed SSR loci and allele sizes, while the vertical axis showed relative peak heights. After initial differentiation, the remaining three (as indicated by the black dashed frame) were further identified by Ip11. IpHT1 (indicated by the green five-pointed star) was identified by Ip06 (184), demonstrating its molecular specificity.

Lastly, Figure 3 comprehensively summarized the SSR identification results (Figures S1–S3, and Table 4) for 16 *I. polycarpa* samples, constructing an SSR fingerprint map. Each accession was uniquely identified by SSR allele sizes, eliminating synonymy confusion and facilitating research on polyploid induction. This SSR fingerprint map not only aided in the identification of each accession but also demonstrated the molecular specificity of IpHT1, highlighting the power and precision of SSR analysis in genetic research.

### 2.3. Inducing Polyploidy

IpHT1, with molecular specificity, high oil content, and ornamental fruits, was chosen for mutation breeding. To achieve this, seeds were treated via tissue culture. After being sterilized, the seeds were placed in a medium containing an inducer. Initially, a comparison was made between the mixed culture and soaking methods regarding their effect on germination rates, as illustrated in Figure S4. The results showed that soaking in sterile water or colchicine (ranging from 0.05% to 0.50%) resulted in lower germination rates (32.33% to 77.67%) compared to tissue culture (77.00% to 93.00%). Furthermore, adding colchicine directly to the medium was found to be more effective in improving germination rates than soaking. Taking into comprehensive consideration the three factors of soaking time, inducer concentration, and breeding conditions on the seeds’ germination, a seed induction system for *I. polycarpa* was established. This system took into account soaking time, inducer concentration, and breeding conditions, with seeds being inoculated into MS medium with chemical inducers via medium addition at a temperature of 25 ± 1 °C, a humidity of 70 ± 5%, and a light intensity of 1500–2000 Lx.

**Figure 4:**
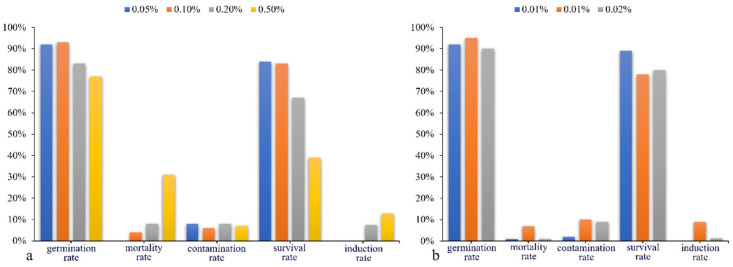
Induction results of colchicine and trifluralin under tissue culture. (**a**) shows colchicine’s effects with concentrations of 0.05% (blue), 0.10% (brown), 0.20% (gray), and 0.50% (yellow). (**b**) displays trifluralin’s effects at 0.005% (blue), 0.01% (brown), and 0.02% (gray). Axes indicated germination, mortality, contamination, survival, and induction rates.

As shown in Figure 4a, under “colchicine + tissue culture”, *I. polycarpa* seeds had germination rates, mortality rates, contamination levels, survival rates, and induction rates. Specifically, optimal colchicine concentrations (0.05% to 0.10%) yielded high germination (92% to 93%) and survival rates (83% to 84%) but did not result in any induction. However, higher concentrations (0.20% to 0.50%) reduced germination and survival but induced mutations (7.50% to 12.80%). Furthermore, as illustrated in Figure 4b, under “trifluralin + tissue culture”, are presented the germination, mortality, contamination, survival, and induction rates. Notably, trifluralin at a concentration of 0.01% induced mutations (9.00%) but also resulted in the highest mortality rate (7%). Despite this, the overall germination rate remained high (90% to 95%), and the survival rate was within a satisfactory range (78% to 89%).

In conclusion, the established seed induction system for *I. polycarpa*, taking into account various factors such as soaking time, inducer concentration, and breeding conditions, provided a platform for effective mutation breeding of IpHT1.

### 2.4. Identification of Mutants

Sixty days post-inoculation, morphological observations of 520 surviving mutagenized *I. polycarpa* seedlings revealed variations. Due to strict aseptic requirements, only preliminary screenings were performed. Figure S5 displays mutagenized plants under identical conditions, highlighting minor differences in some cases (Figure S5b,c) compared to control (Figure S5a) and significant differences in others (Figure S5d–f), which exhibited shorter heights, thicker stems, and delayed leaf growth. These differences were attributed to the inducer inhibition, which led to reduced growth rates and thicker stems. Ultimately, 401 morphologically variant seedlings were selected for further analysis.

Furthermore, to identify mutants among the selected seedlings, FCM was employed. This was performed separately for colchicine-induced (Group A) and trifluralin-induced (Group B) variant seedlings, using uninduced diploid seedlings as controls. Figure S6 demonstrated the ploidy-level peak effects observed during the FCM analysis. The DNA fluorescence content during the G1 phase was measured, and the control samples exhibited peak values of 18–20 w, averaging 194,068.911 (Table S2). Figure S6 and Table S3 show the mutagenized seedlings’ fluorescence peak values and ploidy. As expected, diploid, triploid, and tetraploid plants showed increasing multiples in peak values, with triploids having peaks at 27–30 w, tetraploids at 36–40 w, and chimeric diploid-tetraploid plants showing peaks at both 20 w and 40 w. Through FCM, 50 suspected polyploids (15 triploids and 35 tetraploids) and 3 chimeric plants were identified. Variation coefficients were within 10%, ensuring credibility.

**Figure 5:**
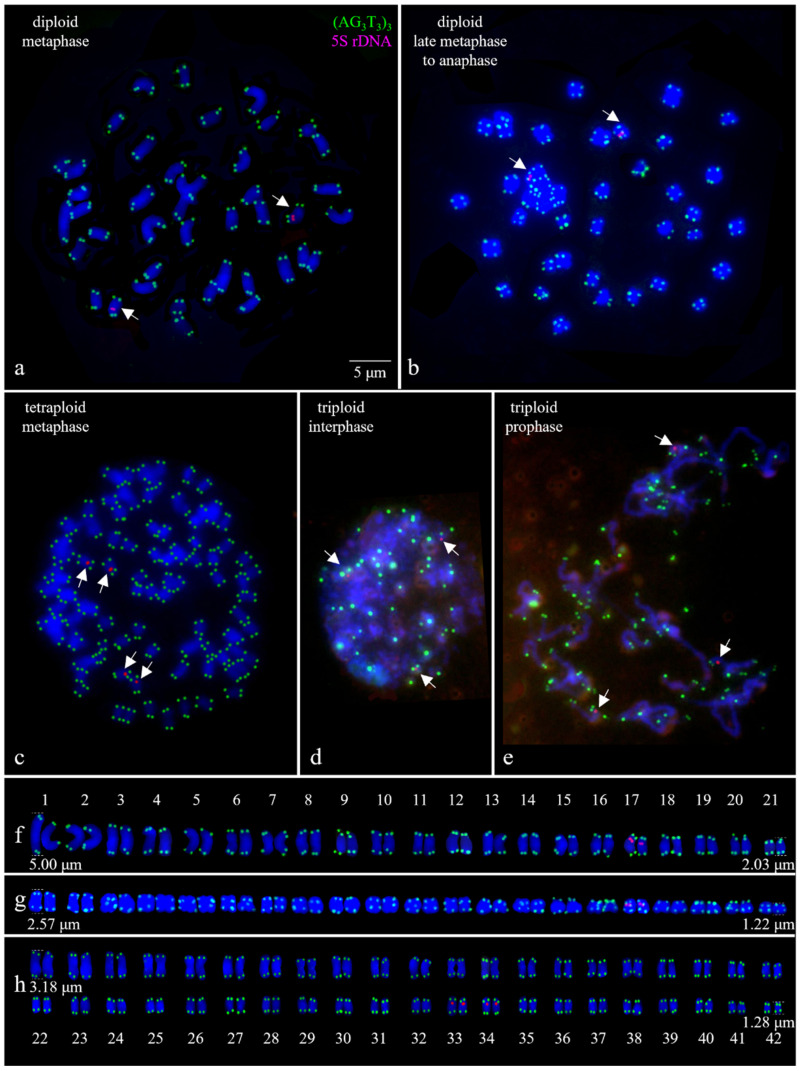
FISH analysis of *I. polycarpa* chromosomes: green [FAM-labeled (AG_3_T_3_)_3_] and red (TAMRA-labeled 5S rDNA) signals on DAPI-stained blue chromosomes. The white arrows in the (**a**–**e**) indicate the 5S rDNA signals. Scale: 5 μm. (**a**,**b**): diploids, (**c**): tetraploids, (**d**,**e**): triploids. (**a**,**c**): metaphase; (**b**): late metaphase; (**d**): interphase; (**e**) prophase. (**f**–**h**): segmented images showing chromosome lengths and pair numbers.

Based on the FCM results, karyotype analysis was then conducted on diploid, suspected triploid, and tetraploid *I. polycarpa*. Using oligonucleotide sequence (AG_3_T_3_)_3_, (GAA)_6_, and 5S rDNA as FISH probes, chromosomes were analyzed in detail. A combined map (Figure 5) was created, featuring the (AG_3_T_3_)_3_ and 5S rDNA signals, with no (GAA)_6_ signal. Mitotic chromosomes were identified for diploids (Figure 5a,b) and tetraploids (Figure 5c), but not triploids, due to a lack of mid-phase cells; instead, interphase (Figure 5d) and prophase (Figure 5e) images were shown. The (AG_3_T_3_)_3_ appeared at chromosome ends, enabling counting (42 for diploids, 84 for tetraploids). 5S rDNA was located proximally, with two signals in diploids and four in tetraploids; triploids showed three signals. The chromosomes were rearranged by length for further analysis (Figure 5f–h). In the diploid metaphase, chromosomes ranged from 2.03 to 5.00 μm in length, with 5S rDNA in a specific pair (33 and 34). In late metaphase, the lengths shortened to 1.22 to 2.57 μm, but the 5S rDNA was still present. Tetraploid metaphase chromosomes were shorter (1.28 to 3.18 μm), with 5S rDNA located in pairs 65/66 and 67/68. However, due to unclear centromere positions, in-depth karyotype analysis was not feasible. Instead, chromosome sizes were qualitatively described based on measurements of the longest and shortest chromosomes. This comprehensive analysis provided valuable insights into the genetic and chromosomal changes induced by the mutagens in *I. polycarpa* seedlings.

### 2.5. Observation of Morphological Characteristics of Identified Mutants

**Figure 6:**
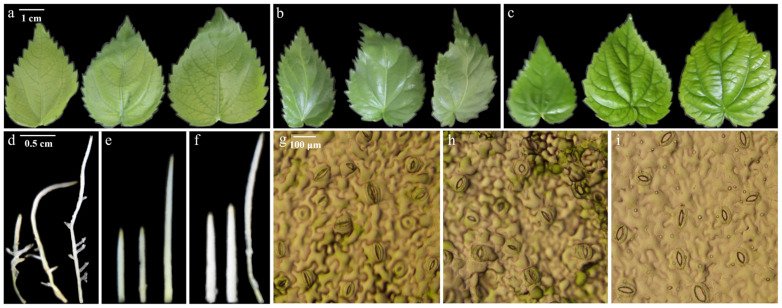
Morphology of *I. polycarpa* leaves, root tips, and stomata at different ploidy levels: diploid (**a**,**d**,**g**), triploid (**b**,**e**,**h**), and tetraploid (**c**,**f**,**i**). Scales: 1 cm (**a**–**c**), 0.5 cm (**d**–**f**), 100 μm (**g**–**i**).

A total of 18 polyploid *I. polycarpa* (7 triploids, 11 tetraploids) were identified using morphology, FCM, and FISH. Morphological comparisons were made among diploid, triploid, and tetraploid mutants at the same growth stage (Figure S7). Furthermore, after 50 days, diploid leaves were light green with fine veins, triploid leaves were bright green with prominent veins, and tetraploid leaves were dark green with darker, sparse veins (Figure 6a–c). Additionally, diploid leaves showed the greatest variability in length, width, and shape index (L/W), with averages of 4.25 cm length, 2.79 cm width, and 1.53 L/W (Figure S8(A1–A3)). In contrast, triploid and tetraploid leaves were shorter (*p* < 0.05) but similar in width and shape (*p* > 0.05), all oval (L/W < 2). Root tips were slender in diploids and robust in triploids and tetraploids (Figure 6d–f).

Moreover, leaf tissues of *I. polycarpa* at different ploidy levels showed stomatal and guard cell differences (Figure 6g–i and Figure S8(B1–B5)). Specifically, stomatal lengths were 28.54 μm (diploid), 40.84 μm (triploid), and 53.36 μm (tetraploid); widths were 12.45 μm (diploid), 12.48 μm (triploid), and 22.35 μm (tetraploid); densities were 27.10, 18.90, and 17.00 pcs/mm^2^, respectively. Triploid stomatal length increased by 43.27% vs. diploid (*p* > 0.05 in width); tetraploid stomata were significantly larger (*p* < 0.05), with 86.97% longer and 79.52% wider stomata. Guard cell lengths were 71.31 μm (diploid), 77.55 μm (triploid), and 99.27 μm (tetraploid); widths were 54.93 μm (diploid), 49.75 μm (triploid), and 63.28 μm (tetraploid). Although guard cell size did not differ significantly between diploid and triploid (*p* > 0.05), tetraploid guard cells were 39.21% longer and 15.20% wider (*p* < 0.05). Stomatal density decreased as ploidy and stomatal size increased. Triploid stomata were elongated, while tetraploid stomata and guard cells were larger, lowering density. Additionally, chlorophyll content in *I. polycarpa* of different ploidy levels is shown in Figure S8(C1–C3). Specifically, diploids, triploids, and tetraploids had chlorophyll a at 0.344, 0.303, and 0.625 mg/g; chlorophyll b at 0.104, 0.097, and 0.186 mg/g; and total chlorophyll at 0.448, 0.400, and 0.811 mg/g, respectively. While triploids showed slight decreases in chlorophyll content, these were not significant (*p* > 0.05). However, tetraploids had significantly higher chlorophyll content (*p* < 0.05), with increases of 81.67% in chlorophyll a, 78.85% in chlorophyll b, and 81.03% in total chlorophyll.

In summary, polyploid *I. polycarpa* (triploids, tetraploids) had thicker stems/roots, fewer lateral roots, larger/darker leaves (ovate shape unchanged), larger stomata/guard cells (reduced density), and significantly higher chlorophyll in tetraploids compared to diploid plants.

## 3. Discussion

### 3.1. Selection of Mutagenesis Materials

To obtain superior polyploids, it is essential to select parents with excellent traits. IpHT1, with 38.5% fruit oil and red color, stands out. Furthermore, genetic analysis of 16 *I. polycarpa* samples from Sichuan showed IpHT1′s distinctiveness. Given its high oil content, ornamental value, and specificity, IpHT1 is ideally suited for mutagenesis breeding. Studies have found rich trait variation in *I. polycarpa* [8,54,55], further supporting this diversity. In this study, the four materials from Jintang County, namely IpHT1, IpJT2, IpJT3, and IpJT4, exhibited rich variation in fruit color and high oil content (IpHT1, IpJT2, and IpJT3). They also have close genetic distances (IpHT1, IpJT3, and IpJT4), except for IpJT2. This indicates that the *I. polycarpa* materials from Jintang County have great potential and should be further screened as potential resources for breeding. Moreover, Li et al. [30] found rich genetic variation in *I. polycarpa* var. *vestita* from four distribution areas in China, consistent with the findings of this study. As a dioecious species with low inbreeding [56], *I. polycarpa*’s allelic heterozygosity contributes to its genetic diversity.

Additionally, genetic distance reflects similarity between individuals [57]. Typically, materials from nearby origins often have closer genetic distances due to domestication [58], albeit with existing variation within regions. However, although the UPGMA clustering results of this study have a certain correlation with geographical distribution, *I. polycarpa* materials from different regions are intermixed and not strictly clustered according to their geographical origin. This observation is consistent with the research results of Jian et al. [59] but differs from the findings of studies on *I. polycarpa* germplasm resources by Li et al. [30]. This intermixing may result from shared ancestry or gene flow during cultivation [60]. In addition, human activities like selection cause gene introgression [61], thereby increasing genetic variation. According to Hamrick’s theory, there is a strong relationship between geographic range and genetic diversity [62]. Consequently, the genetic variation in *I. polycarpa* exhibited a certain regionality, with materials from the same or similar geographical origins having closer genetic distances. This indicated that SSR molecular markers can effectively distinguish resources from different geographical origins. Nevertheless, for a small number of materials, the geographical distance is contrary to the genetic distance. This may be because the genetic relationship of *I. polycarpa* is not completely restricted by geographical location; alternatively, it may be related to human selection activities that have led to gene flow and introgression among the populations of *I. polycarpa* collected from various regions in this study [60].

Furthermore, constructing DNA fingerprint maps using SSR markers reveals *I. polycarpa*’s molecular specificity. Currently, research on DNA fingerprint maps of *I. polycarpa* is still in its infancy. Early-stage research on *I. polycarpa* fingerprints used 4 primer pairs for 16 samples [63]. These maps transform genetic data into an intuitive digital form, facilitating quick genetic relationship analysis [64]. Importantly, selecting optimal primers to maximize variety distinction with minimal markers is crucial [65]. For instance, Zhang et al. [66] used 11 primers for 18 *Prunus* samples but distinguished only 15, highlighting cost and efficiency issues with numerous markers [67]. Highly polymorphic primers minimize marker use. He et al. [68] found bm487 effective, while Sun et al. [69] distinguished 178 *Solanum* samples with 2 primers. Similarly, Wang et al. [70] used 5 primers for 100 *Triticum* samples. In this study, an efficient primer combination distinguished all 16 *I. polycarpa* samples with 4 pairs, enhancing identification efficiency and cost-effectiveness.

### 3.2. Induction System Construction

This study delved into determining the optimal induction system for *I. polycarpa* seeds by examining three key variables: soaking time, inducer concentration, and breeding conditions. Initially, under carefully controlled conditions (temperature maintained at 25 ± 1 °C, humidity at 70 ± 5%, and light intensity between 1500 and 2000 Lx), seeds were inoculated into MS medium with various inducers. Notably, polyploids were successfully produced using colchicine concentrations of 0.20% and 0.50%, as well as trifluralin concentrations of 0.01% and 0.02%. Among these, the highest induction rate of 12.80% was observed with 0.50% colchicine. However, it is worth noting that there is a scarcity of research reports on similar conditions for polyploid induction in *I. polycarpa*.

Furthermore, studies on various plants have shown differing optimal conditions for colchicine treatment to induce polyploids. For instance, Li et al. [71] found that soaking *Pistacia chinensis* Bunge seeds in 0.2% colchicine for 36 h followed by 0.3% for 12 h achieved a 38.10% induction rate. Chen et al. [45] discovered that culturing *Selenicereus undatus* (Haw.) D. R. Hunt. in 50 mg/L colchicine for 5 days was best for polyploid induction. Similarly, Lin et al. [72] reported an 18.6% induction effect when treating *Broussonetia papyrifera* (L.) L’Hér. ex Vent leaves in 450 mg/L colchicine for 3 days. These examples underscore the plant-specific nature of optimal colchicine treatment conditions. Additionally, when combining colchicine and trifluralin with tissue culture, higher concentrations reduced germination and survival rates, while increasing mortality and induction rates. Notably, infection rates remained consistent across concentrations, suggesting they were unrelated to mutagen concentration. This could potentially be attributed to inadequate sterilization or improper handling during experiments.

Importantly, this study also revealed that, as soaking time increased, the impact of colchicine on seed germination intensified. While appropriate concentrations enhanced germination, long-term, high-concentration treatments were toxic, inhibiting growth or causing death. In line with this, Huang et al. [73] found oryzalin more effective than colchicine in inducing fertile *Lilium* L. gametes, with a peak rate of 15.39% at 0.005%. Similarly, Verma et al. [74] showed that low colchicine doses (0.025% and 0.1%) improved *Nigella sativa* L. growth and yield, but higher doses had detrimental effects. In conclusion, the toxicity of chemical mutagens to seeds in this study increased with the duration and concentration of treatment, leading to delayed seed emergence, reduced emergence uniformity, browning or death of plants [57]. These findings are consistent with previous research, highlighting the importance of carefully selecting and optimizing treatment conditions to achieve successful polyploid induction while minimizing adverse effects on seed germination and plant growth.

### 3.3. Mutant Identification

In this study, 401 morphologically variant seedlings were initially screened, yet only 18 polyploids were confirmed by FCM and FISH. Firstly, morphological screening is not accurate and can only serve as a reference [60]. Although polyploids and diploids may have distinct morphological differences [75], not all morphologically variant plants are polyploids due to the influence of genetic and environmental factors, including light, temperature, water, altitude, and nutrients [76]. For instance, Fernando et al. [77] found that tetraploid eucalyptus grew slower and shorter than diploids, possibly due to early growth distortion from chemical mutagen treatment or low emergence uniformity. Furthermore, the average germination potential and germination rate of untreated IpHT1 seeds were low (11.00% and 48.17%, respectively), indicating significant inconsistency in seed growth, which further complicates morphological screening. Thus, identifying polyploids based on early morphological characteristics can lead to significant errors, as screened variants result from combined environmental and structural factors.

Secondly, the FISH results revealed that, out of 50 seedlings suspected to be polyploids based on FCM, only 18 (7 triploids and 11 tetraploids) were confirmed. FCM results did not fully align with chromosome identification, though typically highly accurate [67,78]. Discrepancies in this study could be attributed to experimental timing, where suspected polyploids might have reverted to diploidy, or technical issues such as leaf tissue chopping for cell suspensions being affected by leaf veins or inadequate cutting, leading to impurity peaks and reduced accuracy. Notably, there were no extant prior FCM studies on *I. polycarpa*, and this study identified optimal dissociation solutions tailored to the plant’s cell structure, which is crucial for accurate results [79].

Furthermore, chromosomes, as genetic carriers, are stable in plant reproduction and evolution, with species-specific counts remaining consistent [80,81]. Ploidy differences directly influence chromosome counts; specifically, *I. polycarpa* had diploid 2n = 42 and tetraploid 4n = 84 counts. Our study showed that chromosome numbers aligned with ploidy [3], and 5S rDNA loci correlated positively with ploidy [82]. Although triploid metaphase counts were not determined, chromosomal polyploidization was inferred [83]. FISH, supported by morphology and FCM, offers reliability, with end probes ensuring accurate counts. However, practical limitations, such as the need for metaphase chromosomes from tender root tips and insufficient mutant plant material, particularly in tissue-cultured seedlings with limited tender roots, resulted in fewer FISH identification results compared to the actual number of mutants.

Lastly, the study observed *I. polycarpa* mutations at two growth stages: seedlings after 60 days on medium (cotyledons) and plants stably grown for 50 days post-transfer (true leaves). Cotyledons, crucial for seedling growth but sensitive to mutagenesis, showed delayed growth. True leaves, involved in photosynthesis, were chosen for observation and chlorophyll detection due to reduced variation post-proliferation. Polyploid *I. polycarpa* had thicker stems/roots, larger/darker leaves, larger stomata/guard cells, lower stomatal density, and significantly increased chlorophyll content. These traits enhanced photosynthetic capacity, promoted organic synthesis, and affected growth, aligning with findings from other polyploid studies [84,85]. Comparisons between diploids and polyploids revealed polyploid traits like dwarfism, increased leaf area, chlorophyll, and stress resistance [36,46,86]. Notably, polyploids of *Linum* L. and *Stevia rebaudiana* Bertoni exhibited increased medicinal and market value [87,88], highlighting the potential significance of polyploidy in various plant species.

## 4. Materials and Methods

### 4.1. Experimental Materials

The study utilized 16 varieties (lines) of *I. polycarpa*, as detailed in Table 5. These varieties comprised two specific cultivars: IpHT1, also known as ‘Haitong No. 1’, and IpZF36, or ‘Zaofeng No. 36’. The remaining 14 samples represent elite lines within the species. Notably, among these lines, IpDY1, IpDY2, IpDY3, and IpDY4 belonged to the variety *I. polycarpa* var. *vestita*. The rest of the materials were all classified under *I. polycarpa* var. *polycarpa*, which is abbreviated as *I. polycarpa* for simplicity. All 16 samples were geographically sourced from five districts and counties in Sichuan Province, China: Dayi, Jiajiang, Jintang, Leshan, and Chongzhou. These materials were cultivated at the Yangma Base in Chongzhou, Chengdu City, Sichuan Province. Additionally, some of the cultivation and research activities may have also been conducted at the Chengdu Academy of Agriculture and Forestry Sciences.

### 4.2. Experimental Methods

#### 4.2.1. SSR

As depicted in Figure S9, leaves from 16 *I. polycarpa* samples were preserved in silica gel for SSR analysis. Genomic DNA was extracted using a modified CTAB (hexadecyl trimethyl ammonium bromide) method [89]. SSR primers referenced from Li et al. [30] were synthesized by Sangon Biotech. Co., Ltd. (Shanghai, China). PCR also followed Li et al.’s protocols [30]. GeneMapper4.0 software analyzed capillary electrophoresis results, and Microsoft Excel 2021 compiled genotype data, with homozygous loci as X (where X is the numerical value of the peak at that locus) and heterozygous as X/Y (where X and Y are the numerical values of the two distinct peaks at that locus).

(i) Analysis of genetic diversity parameters: Genetic diversity parameters, including the number of alleles (Na), effective number of alleles (Ne), observed heterozygosity (Ho), expected heterozygosity (He), polymorphism information content (PIC), and Shannon’s information index (I), were calculated for each SSR primer pair using GenALEx6.502 and PowerMarker3.25 software [90]. (ii) Genetic distance analysis: The genetic distance (Gene diversity, GD) between samples was computed using PowerMarker3.25 software. Subsequently, a dendrogram was constructed based on the unweighted pair-group method with arithmetic means (UPGMA) in MEGA 11 software package [91]. Additionally, to further assess the genetic diversity of the 16 samples of *I. polycarpa*, Principal Component Analysis (PCA) was conducted using the R software package (R version 4.2.2) [92]. This PCA took into account factors such as fruit oil content, fruit three-diameters, and the previously computed SSR genetic distance. (iii) Construction of fingerprint profiles and cultivar discrimination: Following Botstein’s theory [63], a primer combination approach was adopted to differentiate the *I. polycarpa* materials under investigation and to construct plant DNA fingerprint profiles. The primer with the highest polymorphism ranking was initially used for discrimination. If not, and all cultivars (lines) could not be distinguished, an additional primer was sequentially included until all materials were uniquely identified.

#### 4.2.2. Polyploid Induction

IpHT1 seeds were threshed and stored at room temperature. They were wrapped in sandpaper, soaked in detergent water to remove the waxy coat, rinsed, soaked in 1.5% potassium dihydrogen phosphate for 24 h, disinfected with 0.5% potassium permanganate for 2 h, rinsed, and soaked in 200 mg/L gibberellic acid at 30 °C for 8 h, then rinsed again.

Immersion method: The pretreated IpHT1 seeds were immersed in colchicine solutions with concentrations of 0.05%, 0.1%, 0.2%, and 0.5%, and sterile water (as a control) for polyploidization. The immersion durations were set at 12, 24, 36, and 48 h. After immersion, the seeds were rinsed with sterile water 4–5 times and placed on filter paper beds in Petri dishes. Each treatment included 100 seeds with three repetitions. The seeds were then placed in a light incubator (humidity 80%, 12 h of light and 12 h of darkness, light intensity 15,000 Lx). Microsoft Excel 2021 was used to record the number of germinated seeds (defined as the emergence of the radicle through the seed coat), and seed germination indices were calculated [93]: Seed germination potential (%) = (Number of germinated seeds on the 7th day/Total number of tested seeds) × 100%, Germination rate (%) = (Total number of germinated seeds on the 10th day/Total number of tested seeds) × 100%, and Germination index (GI): GI = Σ(GT/DT) (where GT is the number of germinated seeds on day T, and DT is the corresponding day).

The tissue culture method involved placing pretreated seeds in sterile tea bags and disinfecting them in a laminar flow hood with 75% ethanol, followed by rinsing and soaking in either 2% sodium hypochlorite for 30 min or 0.1% mercuric chloride for 10 min. Colchicine and trifluralin were added to the culture medium to induce chromosome doubling in *I. polycarpa*. Both chemicals were purchased from Chengdu Haobo You Technology Co., Ltd. Filtered stock solutions of these chemicals were injected into unsolidified medium and thoroughly mixed. Each flask contained medium with varying concentrations of colchicine (0.05–0.5%) [94] and trifluralin (0.005−0.02%) [95]. Prepared media were kept in the dark for 3 days post-autoclaving before inoculation. Seeds were then inoculated onto MS medium supplemented with growth regulators and the selected chemicals (MS + 0.2 NAA + 0.2 6-BA + 0.1 TDZ + 0.3 AC). After 60 days, seedlings were transferred to rooting (1/2 MS + 0.2 NAA + 0.1 6-BA + 0.5 AC) and proliferation (1/2 MS + 0.8 IBA + 0.5 AC) media following ploidy verification. Cultures were maintained in the dark under specific conditions for 5 days before being exposed to light (1500–2000 Lx). Growth parameters, including germination (number of germinated seeds/total inoculated seeds × 100%), mortality (number of dead plants at 60 days/total inoculated seeds × 100%), contamination (number of contaminated plants at 60 days/total inoculated seeds × 100%), and survival (number of surviving plants at 60 days/total inoculated seeds × 100%) rates, were recorded and calculated using Microsoft Excel 2021.

#### 4.2.3. FCM

In accordance with Huang et al. [96], approximately 0.2 g of tender leaves from *I. polycarpa* seedlings (both non-doubled and mutagenized tissue-cultured) were processed in an ultraclean workbench. After vein removal, leaves were placed in a Petri dish, dripped with 1 mL of dissociating solution, and chopped to prepare nuclear suspensions for FCM. The non-doubled seedlings served as the control, using Tris-MgCl_2_, PI, and WPB solutions for detection, each repeated three times. The optimal dissociating solution was determined by its ability to yield a stable G1 peak value in 10 repetitions of the control group, establishing a baseline for mutagenized seedlings. Mutagenized seedlings were then analyzed using this solution, ensuring synchronized detection times and consistent steps between control and test samples.

After a 20 min stand, 60 μL RNAse and 120 μL PI staining solution were added to the cell suspension. The Accuri C6 flow cytometer measured nuclear DNA content. Peaks were saved when cell counts exceeded 5000 and CV was <10%. Excel 2021 calculated G1 peak and CV values. Comparing test and control G1 peaks inferred mutagenized strain ploidy. The FCM graph plots DNA amount (fluorescence intensity) vs. nucleus count. Consistent nucleus numbers per sample ensured accuracy. CV indicates FCM precision; lower CVs reflect better nuclear integrity. CV < 10% yields ideal data; CV < 5%, best results [97]. Test sample ploidy was calculated by dividing its G1 peak intensity by the control’s, then multiplying by the control ploidy. FCM screened suspected doubled *I. polycarpa* for further analysis.

#### 4.2.4. FISH

Newly grown root tips, 1–2 cm in length, were collected from diploids of *I. polycarpa*, wiped clean of soil, and placed in centrifuge tubes. Similarly, root tips of 1–2 cm in length from non-doubled tissue-cultured seedlings and mutagenized tissue-cultured seedlings were separately placed in centrifuge tubes within a super clean workbench. The chromosomes were fixed through a 4 h treatment with Nitrous oxide (N_2_O) and a 5 min treatment with glacial acetic acid, followed by storage in 75% ethanol at −20 °C. Chromosome slide preparation for the root tips was performed according to the method described by Luo et al. [52], facilitating subsequent hybridization with probes. The probes used in the experiment, including (AG_3_T_3_)_3_, 5S rDNA, and (GAA)_6_, were synthesized by Sangon Biotech Co., Ltd. (Shanghai, China). The fluorescent signals of the probes were hybridized onto the chromosomes on the slides, following the specific experimental steps outlined by He et al. [53].

Chromosomes on the hybridized slides were tracked utilizing the Olympus BX-63 microscope, manufactured by Olympus Corporation in Tokyo, Japan, and FISH images were captured with the DP-70 CCD camera designated for use with the microscope. The FISH images were processed using Adobe Photoshop 2021 (Adobe Systems Inc., San Jose, CA, USA) for chromosome extraction. The original images were cropped, legends were added, and the lengths of the metaphase chromosomes were measured using Image J software (version 1.8.0.345). Chromosome counting was performed based on the distribution of the (AG_3_T_3_)_3_ probe at the ends of chromosomes. The number and distribution of sites of oligonucleotide probes 5S rDNA and (GAA)_6_ on the chromosomes of mutagenized and control plants were statistically compared. Based on the FISH results, the number of polyploids was counted.

#### 4.2.5. Measurement of Morphological and Physiological Indices

Five plants from diploid, triploid, and tetraploid *I. polycarpa* tissue-cultured seedlings were randomly selected. Leaf length and width (middle lobe) were measured to 0.01 cm accuracy using a ruler. The leaf shape index [98] (L/W = leaf length/leaf width) was calculated. Leaves were classified as lanceolate (L/W > 3), long elliptical (2.5 < L/W < 3), elliptical (2 < L/W < 2.5), or ovate (L/W < 2). SPSS 24.0 was used for one-way ANOVA and Origin 2021 for boxplots. Additionally, 5 plants from diploid, triploid, and tetraploid sets were randomly selected. For each, the lower epidermis was torn off from 4 similar-sized leaves. Each was placed on a slide with water, covered with a coverslip, and observed under a 20× microscope. Guard cell and stomata dimensions and counts were recorded, and stomatal density was calculated [99]. SPSS 24.0 was used for ANOVA and Origin 2021 for boxplots.

Chlorophyll was extracted from *I. polycarpa* tissue-cultured seedlings using the ethanol method [100]. Five plants from diploid, triploid, and tetraploid sets were randomly selected. Leaf strips (0.1 cm width) from the top leaf of each plant were soaked in 95% ethanol to obtain chlorophyll extract, then stored in the dark. The extract’s absorbance was measured using a spectrophotometer, with four replicates per sample. Chlorophyll a, b, and total contents were calculated using Arnon’s formula [101], adjusted for leaf area. Data were analyzed using Excel 2021, and ANOVA was performed using SPSS 24.0. Boxplots were generated using Origin 2021.

## 5. Conclusions

Based on genetic diversity analysis of 16 *I. polycarpa* varieties (lines) from Sichuan, IpHT1 was chosen for polyploid induction via tissue culture and chemical inducers. Morphology, FCM, and FISH identified mutant ploidy. Our findings enhance *I. polycarpa* selection and breeding efficiency. However, geography, primers, and sample size limited comprehensiveness. New polyploid materials were screened, but numbers were limited. Future work should expand breeding scale, cultivate in the long-term, and observe materials to assess ploidy and genetic stability.

## Figures and Tables

**Table 1:** Fruit traits of *I. polycarpa*.

Accession	Sex	Color	Oil Content	Longitudinal/Transverse/ Lateral Diameter (mm)
IpDY1	monoecism	dull-red	32.10%	8.23/8.66/8.33
IpDY2	female	Red	33.80%	8.36/9.17/9.01
IpDY3	female	Red	31.30%	7.76/8.12/8.03
IpDY4	male	-	-	-
IpJJ1	female	Red	**38.90%**	4.50/4.63/4.67
IpJJ2	female	Red	35.17%	8.14/8.63/8.45
IpJJ3	female	Red	32.08%	**11.36/13.97/12.84**
IpHT1	female	Red	**38.50%**	**10.24/10.85/10.72**
IpJT2	female	red-orange	**38.06%**	7.31/7.62/7.48
IpJT3	female	yellow-orange	37.40%	8.13/8.25/8.04
IpJT4	female	Yellow	34.60%	7.68/7.79/7.82
IpLS1	female	Red	33.41%	6.94/7.29/7.09
IpLS2	female	Red	33.65%	6.64/7.48/7.02
IpLS3	female	Red	34.63%	7.66/9.12/8.16
IpLS4	female	Red	35.78%	8.18/8.35/8.06
IpZF36	female	Red	33.90%	**9.36/9.42/9.41**

Note: The bold text indicates the top three rankings in fruit oil content and fruit size.

**Table 2:** Genetic parameter analysis of polymorphic loci from 11 pairs of primers in *I. polycarpa*.

Primer	Na	Ne	Shannon I	Ho	He	PIC	Fluorochrome
Ip01	4	3.630	1.334	1.000	0.724	0.777	FAM
Ip05	2	2.000	0.693	1.000	0.500	0.568	FAM
Ip06	3	2.571	1.011	1.000	0.611	0.618	HEX
Ip07	8	5.120	1.836	1.000	0.805	0.827	HEX
Ip09	2	2.000	0.693	1.000	0.500	0.419	HEX
Ip11	6	3.765	1.511	1.000	0.734	0.786	ROX
Ip13	2	2.000	0.693	1.000	0.500	0.608	ROX
Ip15	2	2.000	0.693	1.000	0.500	0.511	ROX
Ip16	4	2.970	1.197	1.000	0.663	0.674	TAMRA
Ip17	2	2.000	0.693	1.000	0.500	0.419	TAMRA
Ip18	3	2.571	1.011	1.000	0.611	0.532	TAMRA
Total	38	30.627					
Mean	3.455	2.784	1.033	1.000	0.604	0.613	

**Table 3:** Genetic distances among 16 tested samples of *I. polycarpa*.

	IpDY1	IpDY2	IpDY3	IpDY4	IpJJ1	IpJJ2	IpJJ3	IpHT1	IpJT2	IpJT3	IpJT4	IpLS1	IpLS2	IpLS3	IpLS4	IpZF36
IpDY1	0	-	-	-	-	-	-	-	-	-	-	-	-	-	-	-
IpDY2	0.455	0	-	-	-	-	-	-	-	-	-	-	-	-	-	-
IpDY3	0.455	0.273	0	-	-	-	-	-	-	-	-	-	-	-	-	-
IpDY4	0.409	0.182	0.273	0	-	-	-	-	-	-	-	-	-	-	-	-
IpJJ1	0.318	0.364	0.318	0.364	0	-	-	-	-	-	-	-	-	-	-	-
IpJJ2	0.455	0.318	0.045	0.273	0.318	0	-	-	-	-	-	-	-	-	-	-
IpJJ3	0.500	0.227	0.409	0.318	0.364	0.409	0	-	-	-	-	-	-	-	-	-
**IpHT1**	**0.455**	**0.409**	**0.500**	**0.364**	**0.318**	**0.500**	**0.364**	0	-	-	-	-	-	-	-	-
IpJT2	0.409	0.409	0.318	0.318	0.500	0.318	0.500	**0.545**	0	-	-	-	-	-	-	-
IpJT3	0.500	0.273	0.318	0.227	0.364	0.318	0.364	**0.364**	0.455	0	-	-	-	-	-	-
IpJT4	0.500	0.409	0.364	0.364	0.409	0.364	0.409	**0.409**	0.318	0.273	0	-	-	-	-	-
IpLS1	0.273	0.364	0.318	0.318	0.045	0.318	0.318	**0.273**	0.455	0.364	0.364	0	-	-	-	-
IpLS2	0.500	0.318	0.409	0.364	0.318	0.409	0.455	**0.409**	0.591	0.318	0.455	0.364	0	-	-	-
IpLS3	0.318	0.409	0.364	0.455	0.182	0.409	0.409	**0.364**	0.545	0.455	0.455	0.182	0.409	0	-	-
IpLS4	0.455	0.273	0.273	0.364	0.318	0.318	0.273	**0.364**	0.545	0.318	0.409	0.318	0.364	0.318	0	-
IpZF36	0.273	0.500	0.500	0.455	0.318	0.500	0.545	**0.409**	0.591	0.455	0.500	0.273	0.455	0.364	0.409	0

Note: The data marked in bold are the genetic distances between *I. polycarpa* ‘Haitong 1’ (IpHT1) and other accessions.

**Table 4:** Four SSR primer pairs identified 16 *I. polycarpa* samples.

Identification	Primer	Accession Identified
Firstly	Ip06	IpDY1, IpDY3, IpHT1
	Ip07	IpDY1, IpDY2, IpDY3, IpDY4, IpJJ1, IpJJ2, IpJT2, IpJT3, IpJT4, IpZF36
	Ip11	IpLS4
	Ip18	IpJJ3
Secondly	Ip11	IpLS1, IpLS2, IpLS3

Note: There were two identification stages: first 13 accessions, then the remaining three.

**Table 5:** Sampling information of *I. polycarpa*.

Accession	Variety Name	License Number	Location	Longitude and Latitude
IpDY1	-	-	Dayi, Sichuan	103.52071 E, 30.58759 N
IpDY2	-	-	Dayi, Sichuan	103.52071 E, 30.58759 N
IpDY3	-	-	Dayi, Sichuan	103.52071 E, 30.58759 N
IpDY4	-	-	Dayi, Sichuan	103.52071 E, 30.58759 N
IpJJ1	-	-	Jiajiang, Sichuan	103.57156 E, 29.73753 N
IpJJ2	-	-	Jiajiang, Sichuan	103.57156 E, 29.73753 N
IpJJ3	-	-	Jiajiang, Sichuan	103.57156 E, 29.73753 N
IpHT1	Haitong 1	Chuan R-SC-IP-013-2019	Jintang, Sichuan	104.41205 E, 30.86203 N
IpJT2	-	-	Jintang, Sichuan	104.41205 E, 30.86203 N
IpJT3	-	-	Jintang, Sichuan	104.41205 E, 30.86203 N
IpJT4	-	-	Jintang, Sichuan	104.41205 E, 30.86203 N
IpLS1	-	-	Leshan, Sichuan	103.07879 E, 29.24447 N
IpLS2	-	-	Leshan, Sichuan	103.07879 E, 29.24447 N
IpLS3	-	-	Leshan, Sichuan	103.07879 E, 29.24447 N
IpLS4	-	-	Leshan, Sichuan	103.07879 E, 29.24447 N
IpZF36	Zaofeng 36	Chuan R-SC-IP-002-2019	Chongzhou, Sichuan	104.10194 E, 30.65984 N

Note: IpDY1, IpDY2, IpDY3, and IpDY4 are varieties of *I. polycarpa* var. *vestita*, while the remaining materials are all *I. polycarpa* var. *polycarpa*, abbreviated as *I. polycarpa*.

## Data Availability

The original contributions presented in the study are included in the article/Supplementary Materials, further inquiries can be directed to the corresponding author.

## Supplementary Materials

The following supporting information can be downloaded at: https://www.mdpi.com/article/10.3390/plants13233394/s1, Figure S1: Scatter diagram of 11 SSR loci exhibiting polymorphic distribution during detecting the 16 samples of *I. polycarpa*; Figure S2: Allele amplification size and corresponding fluorescence intensity of *I. polycarpa*; Figure S3: Using four SSR primer pairs, 16 *I. polycarpa* samples were identified by their allele sizes (bold); Figure S4: Germination rates of *I. polycarpa* seeds under different concentrations of colchicine treatment through tissue culture and soaking methods; Figure S5: Morphology of *I. polycarpa* tissue culture seedlings after 60 days of inoculation; Figure S6: Flow cytometry analysis of *I. polycarpa* seedlings; Figure S7: Seedlings of different ploidy of *I. polycarpa* that have grown steadily on proliferation medium for 50 days; Figure S8: Leaf morphology, stomatal characteristics, and chlorophyll content of *I. polycarpa* with different ploidy levels; Figure S9: Sixteen samples of *I. polycarpa* leaves preserved in silica gel; Table S1: Nineteen pairs of primers were used for SSR analysis of 16 samples of *I. polycarpa*; Table S2: Fluorescence peak values diploid (control group) of *I. polycarpa*; Table S3: Fluorescence peak values and ploidy identification of *I. polycarpa* mutations.
