# Supplementary material for: Genetic Diversity Analysis and Polyploid Induction Identification of Idesia polycarpa
Source: Plants (Basel). 2024 Dec 3;13(23):3394. doi: 10.3390/plants13233394 (PMC11644141; doi:10.3390/plants13233394)
Supplement: Supplementary file 1 [file plants-13-03394-s001.zip › plants-3314789-supplementary.pdf]

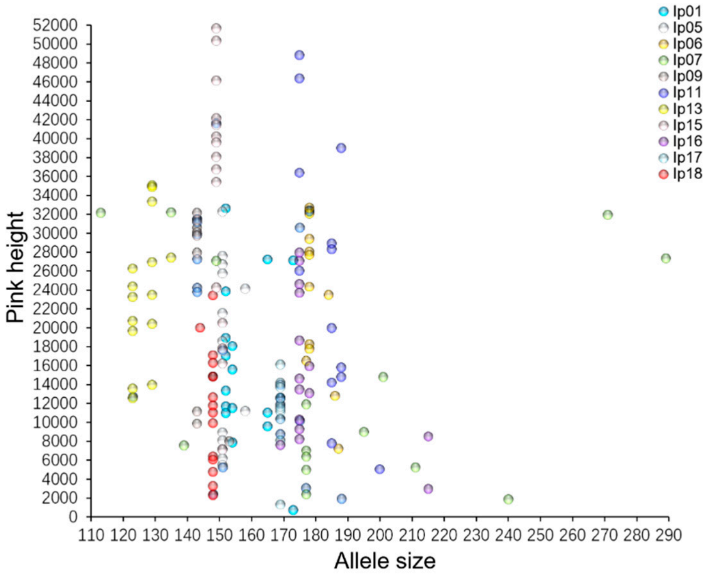

Figure S1 Scatter diagram of 11 SSR loci exhibiting polymorphic distribution during detecting the 16 accessions of *I. polycarpa*. The SSR loci are represented by different colors and symbols. The x-axis indicates allele size, while the y-axis indicates relative peak height. The allele sizes ranged from 113 to 289 nucleotides, while the relative peak heights ranged from 710 to 51,670. Eleven pairs of primers.

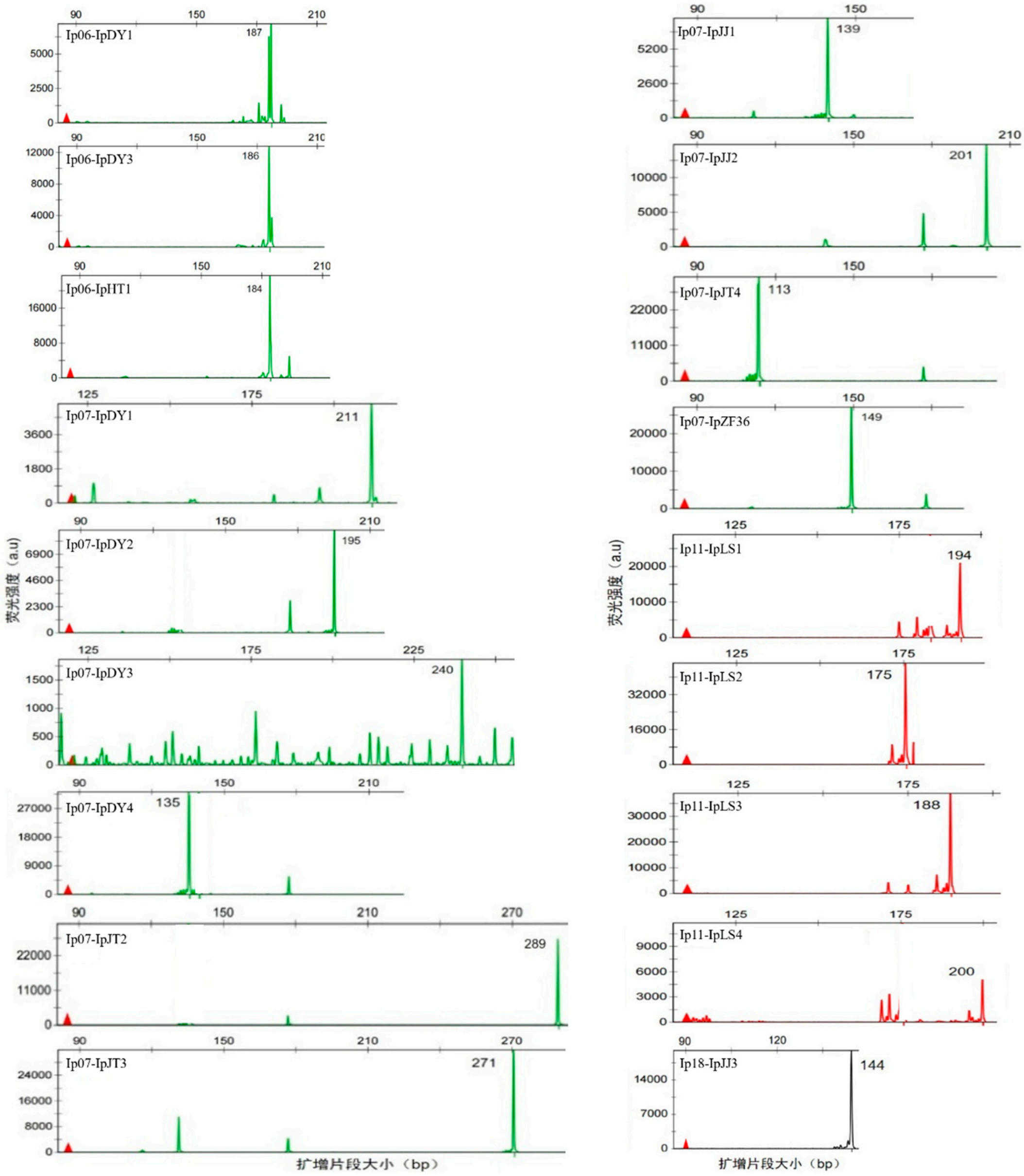

Figure S2 Allele amplification size and corresponding fluorescence intensity of *I. polycarpa*. The size of each allele is labeled near its respective valid peak. The alleles and materials are annotated in the upper left corner of each subgraph.

	IpDY1	IpDY2	IpDY3	IpDY4	IpJJ1	IpJJ2	IpJJ3	IpHT1	IpJT2	IpJT3	IpJT4	IpLS1	IpLS2	IpLS3	IpLS4	IpZF36
Ip18	148	148	148	148	148	148	**144**	148	148	148	148	148	148	148	148	148
Ip06	**187**	178	**186**	178	178	178	178	**184**	178	178	178	178	178	178	178	177
Ip11	188	175	188	175	185	188	175	185	175	185	185	**185**	**175**	**188**	**200**	175
Ip07	**211**	**195**	**240**	**135**	**139**	**201**	177	177	**289**	**271**	**113**	177	177	177	177	**149**

Figure S3 Using four SSR primer pairs, 16 *I. polycarpa* samples were identified by their allele sizes (bold). Thirteen were identified by one primer (black ellipses), and the remaining three by Ip11 after distinguishing the first 13 (purple dashed ellipse).

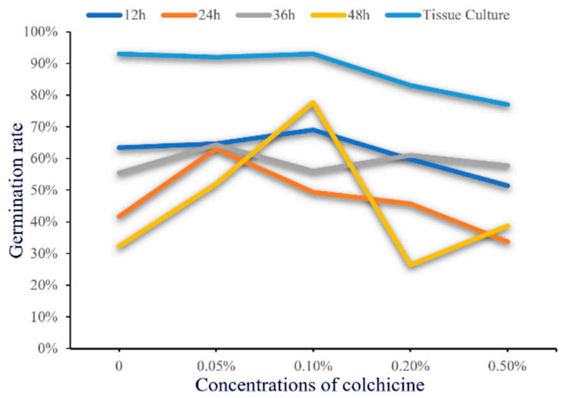

Figure S4 Germination rates of *I. polycarpa* seeds under different concentrations of colchicine treatment through tissue culture and soaking methods. The horizontal axis represents the different concentrations of colchicine. The vertical axis represents the germination rate of *I. polycarpa* seeds. The blue line indicates the germination rate of *I. polycarpa* seeds under tissue culture conditions. The dark blue line represents the germination rate after soaking in colchicine for 12 hours. The brown line represents the germination rate after soaking in colchicine for 24 hours. The gray line indicates the germination rate after soaking in colchicine for 36 hours. The yellow line represents the germination rate after soaking in colchicine for 48 hours.

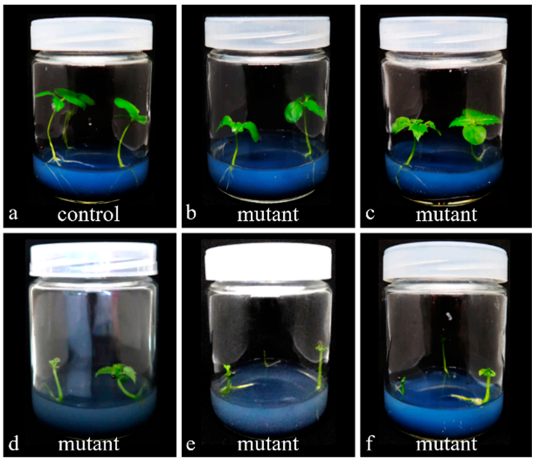

Figure S5 Morphology of *I. polycarpa* tissue culture seedlings after 60 days of inoculation. Figure S5a is the control, and Figures S5b-S5f are the mutagenized seedlings.

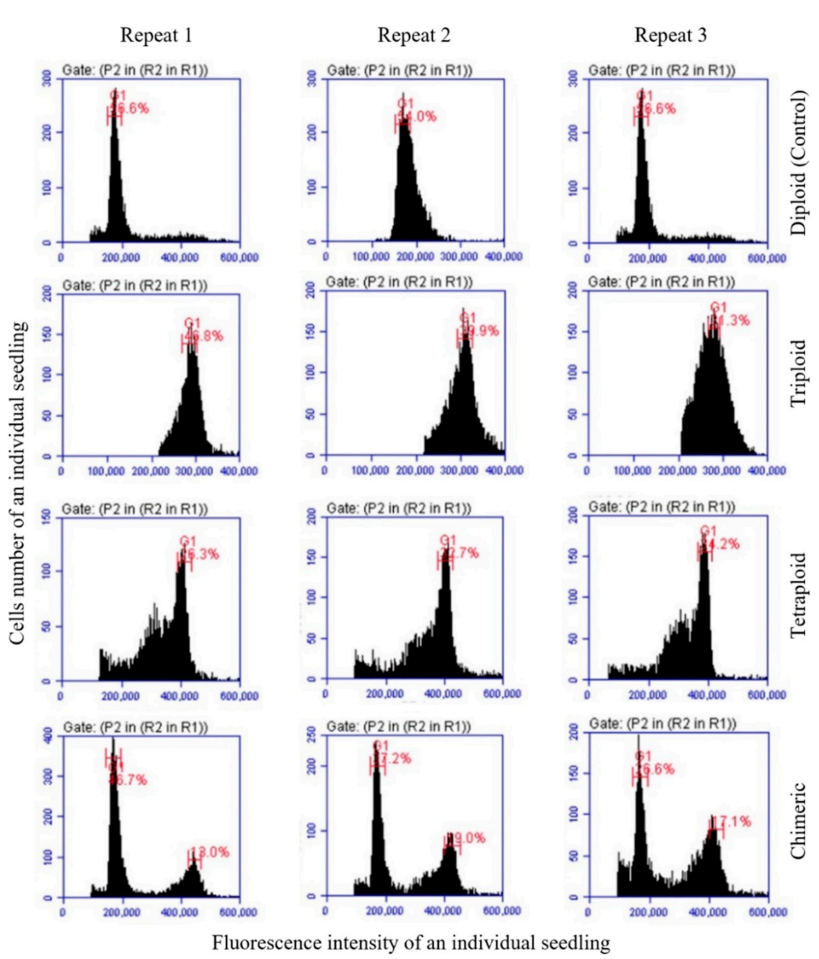

Figure S6 Flow cytometry analysis of *I. polycarpa* seedlings. The horizontal axis represents the fluorescence intensity of an individual seedling being tested, while the vertical axis indicates the number of cells within that same seedling. The diploid (control), triploid, tetraploid, and chimeric plants are each repeated with three individual plants.

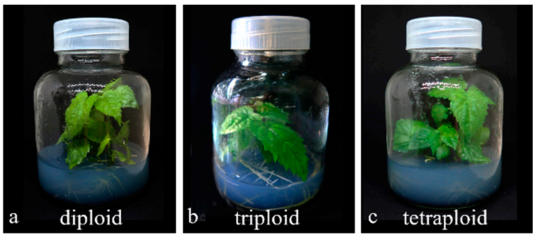

Figure S7 Seedlings of different ploidy of *I. polycarpa* that have grown steadily on proliferation medium for 50 days. Figure S7a is diploid, Figure S7b is triploid, and Figure S7c is tetraploid.

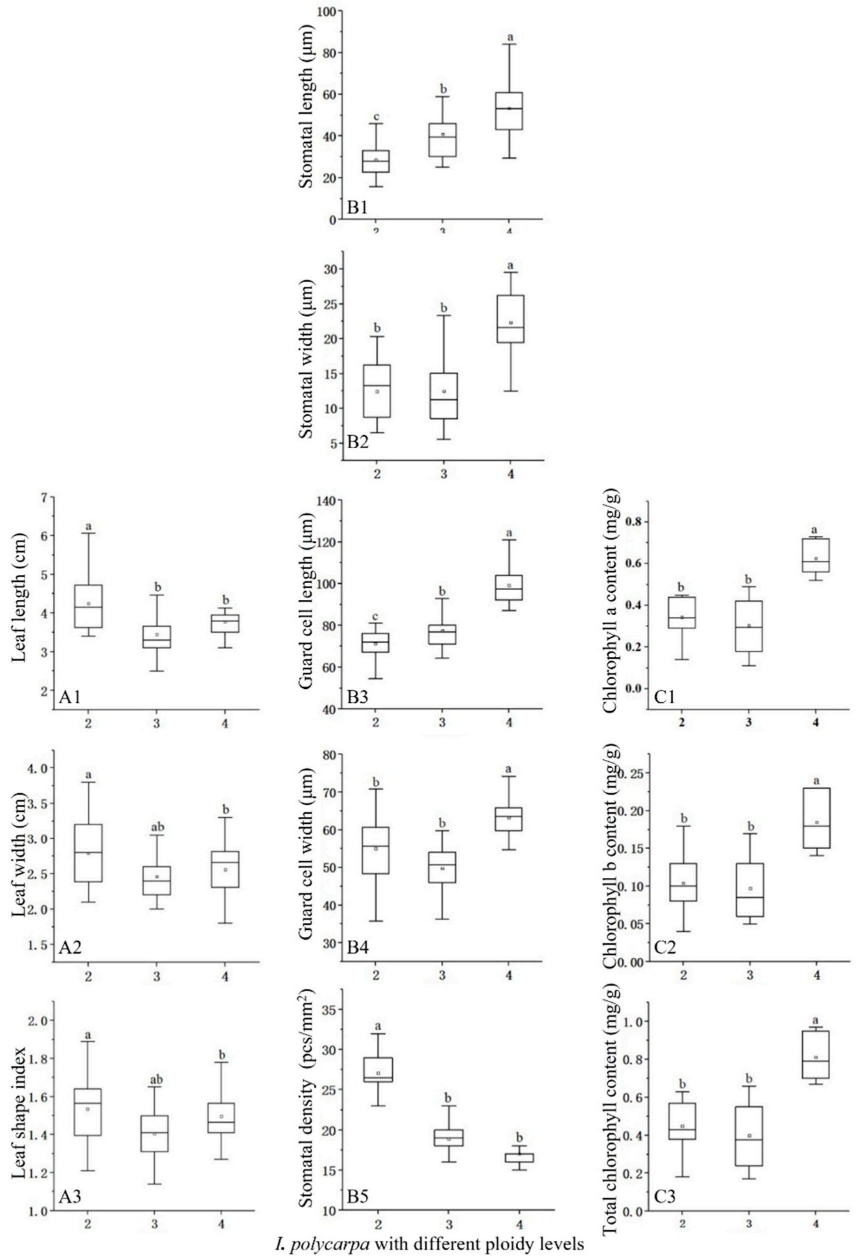

Figure S8 Leaf morphology, stomatal characteristics, and chlorophyll content of *I. polycarpa* with different ploidy levels. Figure S8 (A1-A3) represent leaf length, leaf width, and leaf shape index, respectively. Figure S8(B1-B5) depict stomatal length, stomatal width, guard cell length, guard cell width, and stomatal density, respectively. Figure S8(C1-C3) indicate chlorophyll a content, chlorophyll b content, and total chlorophyll content, respectively. The horizontal axis represents the ploidy levels: diploid, triploid, and tetraploid. The vertical axis represents the leaf morphology, stomatal characteristics, and chlorophyll content.

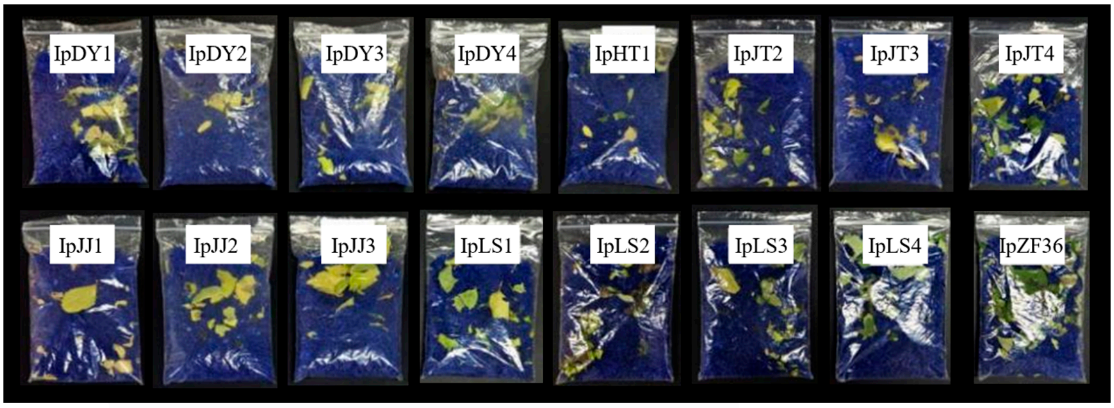

Figure S9 Sixteen samples of *I. polycarpa* leaves preserved in silica gel.

Table S1 Nineteen pairs of primers were used for SSR analysis of 16 samples of *I. polycarpa.*

Primer	Length (bp)	Forward prime (5' to 3')	Reverse primer (5' to 3')
Ip01*	153	TTCCTTCACCAACCCAGTAAGTA	TCCATGCAGATGAAAAGAAAACT
Ip02	157	ACACGCCTCCACATTAGATAAAA	GATTGTCTACGAACAGCATTTCC
Ip03	158	GGACATATCTGACGATTTTCGAG	CCACAACTTCTAGTGAGGTTTGC
Ip04	144	CTTAATATCAGTAGGCCTCGTCG	AGTAGTCAAGAGAGTGGTGGTGG
Ip05*	136	GAACGTGGAGGGAGTTAAAGATT	TTGATCCTTGGACCTCTTATTCC
Ip06*	160	TCTCCATCGTACTATTTGAATCTCAT	ATCAATCAAATCAATCACAAGCC
Ip07*	160	GCAAATTCAAATTTCCTTCCTTC	CAAACTGGGGTCATCTTGGTAT
Ip08	156	TAAGGGAGCATTGAAAGGTAACA	CGTTTCGATACCTTCTTGAACAC
Ip09*	128	CTTCTGAAGCAAATGATCAACCT	GACAAAAGCACTTTCTCCACAAC
Ip10	130	GAAGATCGAAGGTCAACCCTACT	GAGAAGATCTTTGAGAGATGCCA
Ip11*	158	TCCTAATCAACCCTCAACCATC	AGATGAAACTGTGGGGGTGTTAT
Ip12	158	GAAATGCCCATGGTAAACAAGTA	TGCATTAGGATAATTTGATTGTCG
Ip13*	113	ATTTAAAGCAAAACCTCTCCTGC	TCCGTCTTTGTCGATGTATTACC
Ip14	158	CTCAATCTATGCAATACCAAGGC	CGCTTGTACCTAATCCAAAATCA
Ip15*	132	GCCTGAATTGGTTGTTTAGTCTG	AGCTTTGAAGATTTGTATGCAGC
Ip16*	157	TATGCTGCTTCAACTATTCCCAT	GGAGTGGTGTATGATCTCTTGCT
Ip17*	149	GTGGTAGAAATCACAAGCGTAGC	ACTCTCAGCACAATGGACTTTTC
Ip18*	126	GGAGAGGATAGGGAATTTGAAGA	AAATTATGCCCAAAATTACCCTC
Ip19	145	GGTGAAGTTTGTGGATGCTTTAT	AAACTCCAAAATTACCATCACCA

Note: the aforementioned 19 primers used were from Li et al. [30]. Among them, 11 marked with (*) showed polymorphism among 19 *I. polycarpa* samples.

Table S2 Fluorescence peak values diploid (control group) of *I. polycarpa.*

Accession	Fluorescence peak	CV (%)	Ploidy
CK01	189,085.45	5.22	2
CK02	188,638.86	6.90	2
CK03	198,502.07	4.35	2
CK02	196,818.52	5.06	2
CK03	200,316.75	4.98	2
CK04	193,285.98	5.17	2
CK05	192,568.40	5.09	2
CK06	200,832.06	4.96	2
CK07	196,827.58	5.29	2
CK08	198,813.44	4.98	2
Mean	195568.911	5.20	-

Table S3 Fluorescence peak values and ploidy identification of *I. polycarpa* mutations.

Accession	Fluorescence peak	CV (%)	$\frac{\text{Treatment peak value}}{\text{Control peak value}}$	Estimate ploidy
A$_2$63	289,315.39	3.13	1.5	3
A$_2$74	312,041.22	2.92	1.6	3
A$_3$1	280,729.58	2.90	1.4	3
A$_3$4	395,240.43	3.95	2.0	4
A$_3$5	365,728.44	3.93	1.9	4
A$_3$14	371,417.03	3.98	1.9	4
A$_3$16	294,429.05	3.58	1.5	3
A$_3$17	382,220.08	3.31	2.0	4
A$_3$19	296,268.67	3.17	1.5	3
A$_3$20	297,905.52	3.22	1.5	3
A$_3$23	364,325.36	3.59	1.9	4
A$_3$24	368,756.74	3.37	1.9	4
A$_3$25	395,798.94	3.98	2.0	4
A$_3$27	360,107.50	2.71	1.9	4
A$_4$1	402,322.93	4.23	2.1	4
A$_4$2	405,240.43	3.95	2.1	4
A$_4$11	369,111.67	3.82	1.9	4
A$_4$13	275,388.88	4.19	1.4	3
A$_4$14	359,342.13	6.92	1.9	4
A$_4$18	365,628.96	5.84	1.9	4
B1	369,583.43	3.75	1.9	4
B6	368,438.92	3.73	1.9	4
B8	378,956.96	3.34	2.0	4
B12	302,556.34	3.24	1.6	3
B20	371,157.50	6.39	1.9	4
B22	378,540.48	3.26	2.0	4
B29	407,134.11	3.81	2.1	4
B35	365,170.66	2.78	1.9	4
B39	401,132.99	3.18	2.1	4
B42	404,807.18	3.38	2.1	4
B47	380,752.91	7.12	2.0	4
B55	308,059.51	4.06	1.6	3
B57	305,092.52	4.14	1.6	3
B60	312,525.11	3.59	1.6	3
B63	366,099.57	2.56	1.9	4
B66	371,417.03	3.98	1.9	4
B67	408,928.40	2.75	2.1	4
B68	392,702.44	2.87	2.0	4
B76	405,279.47	2.53	2.1	4
B77	387,998.16	3.11	2.0	4
B81	372,850.07	2.45	1.9	4
B84	297,922.58	1.93	1.5	3
B85	171,157.50	6.39	0.9	chimera
	445,279.47	2.53	2.3	
B95	171,711.40	6.73	0.9	chimera
	424,807.18	3.38	2.2	
B97	168,136.56	6.89	0.9	chimera
	421,370.80	3.11	2.2	
B98	401,370.80	3.11	2.1	4
B100	404,699.75	2.90	2.1	4
B104	403,980.89	3.14	2.1	4
B108	337,881.51	2.96	1.7	3
B110	331,682.53	2.99	1.7	3
B111	325,461.82	3.13	1.7	3
B127	461,132.99	3.18	2.3	4
B129	459,111.67	3.82	2.3	4
